# Supplementary material for: Signatures of positive selection reveal a universal role of chromatin modifiers as cancer driver genes
Source: Sci Rep. 2017 Oct 13;7:13124. doi: 10.1038/s41598-017-12888-1 (PMC5640613; doi:10.1038/s41598-017-12888-1)
Supplement: Supplementary file 1 — Supplementary Material [file 41598_2017_12888_MOESM1_ESM.pdf]

Supplementary Material

Signatures of positive selection reveal a universal role of chromatin modifiers as cancer driver genes

Luis Zapata, Hana Susak, Oliver Drechsel, Marc R. Friedländer, Xavier Estivill and Stephan Ossowski

Supplementary Figures

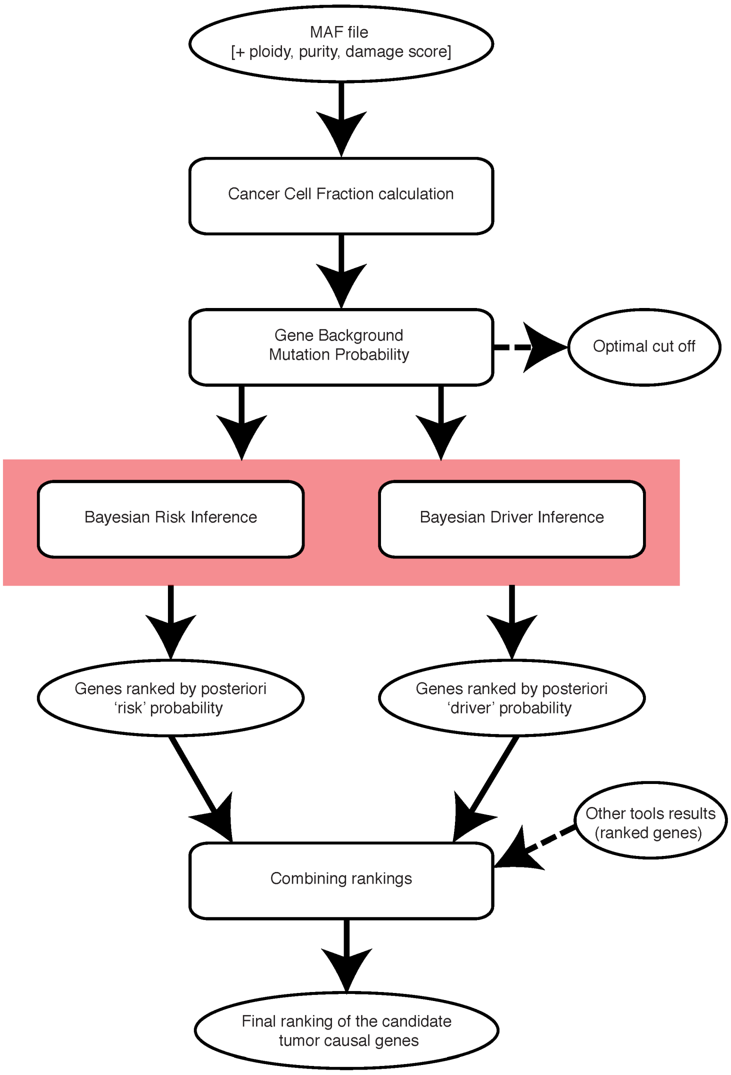

Supplementary Figure 1

cDriver Pipeline. Schematic overview of cDriver's workflow. Input is a standardized MAF file with optional columns: ploidy, purity and functional impact score. In this diagram, ellipsoids represent data or files. Rectangles represent functions or operations. The first step is the calculation of cancer cell fraction. The second step calculates the background mutation

probability using the model described in online methods. The third step calculates the posterior probabilities per gene using two Bayesian models. The final output is a ranking of all genes given by the combination of the previously obtained rankings.

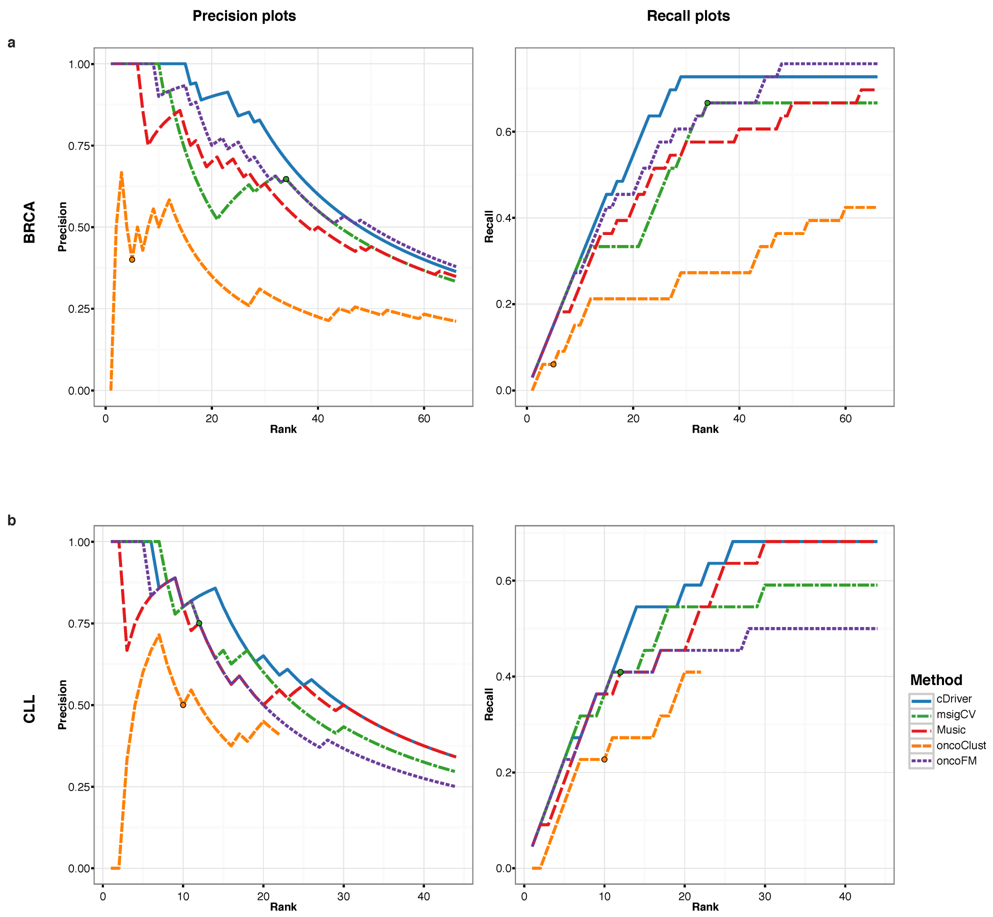

Supplementary Figure 2

Precision and recall for five driver identification methods benchmarked on different datasets. Precision and recall plots for BRCA (**a**), CLL (**b**). Precision and recall are shown for methods: cDriver (blue), MutsigCV (misgCV, green), MuSiC(red), OncodriveFM (oncoFM, purple) and OncodriveCLUST(oncoClust, orange). As gold standard, manually compiled lists of 44 genes for BRCA and 22 genes for CLL were used, while Cancer Gene Census was used for Pancan12.

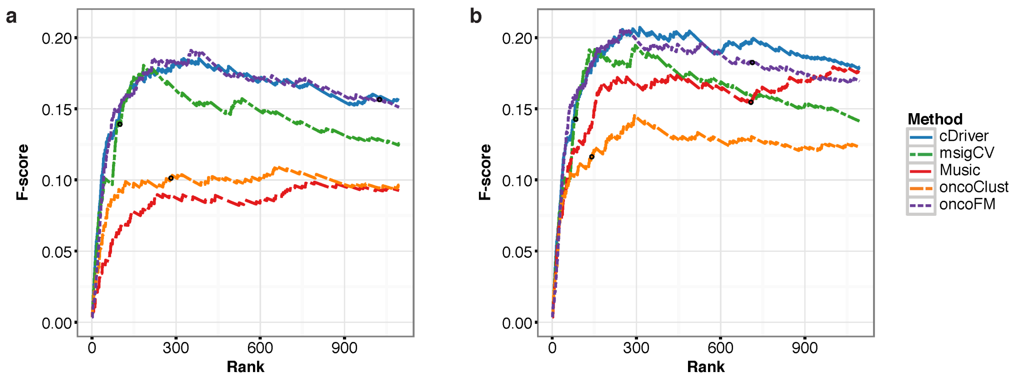

Supplementary Figure 3

F-score measure on filtered versus unfiltered data. F-score curves for competing methods with and without post-filtration of non-expressed genes in the Pancan12 dataset. All methods are shown, and their corresponding significance threshold ranking using Q value < 0.1.

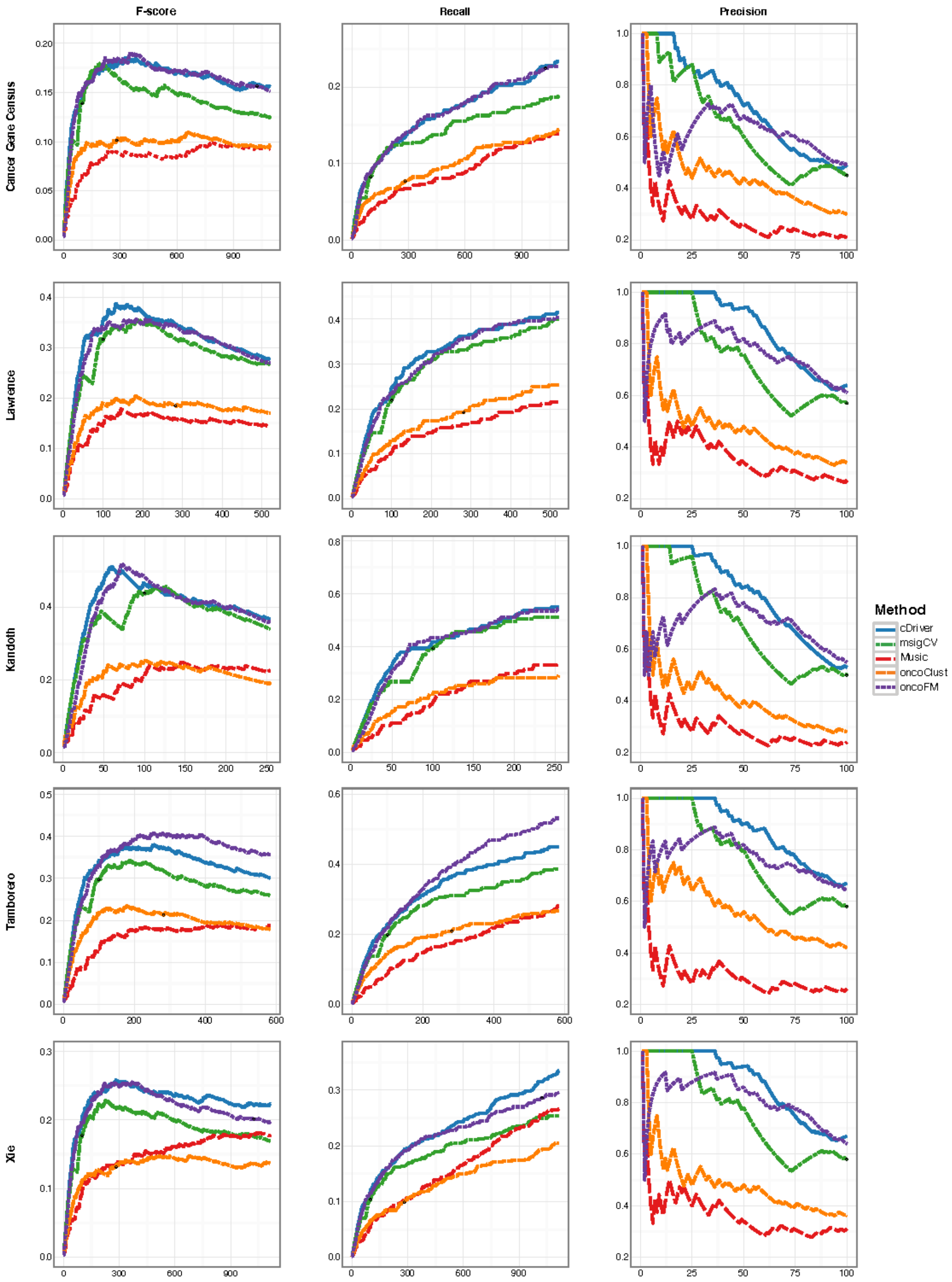

Supplementary Figure 4.

Evaluation of several measures for five driver identification methods benchmarked on Pancan12. Benchmarking of F-score, precision, and recall measures for five driver identification methods benchmarked on Pancan12 across five gold standard datasets[1,2,3,4,5]. X-axis shows the ranked list of genes for each tool. Y-axis shows F-score, Precision, and Recall according to the header.

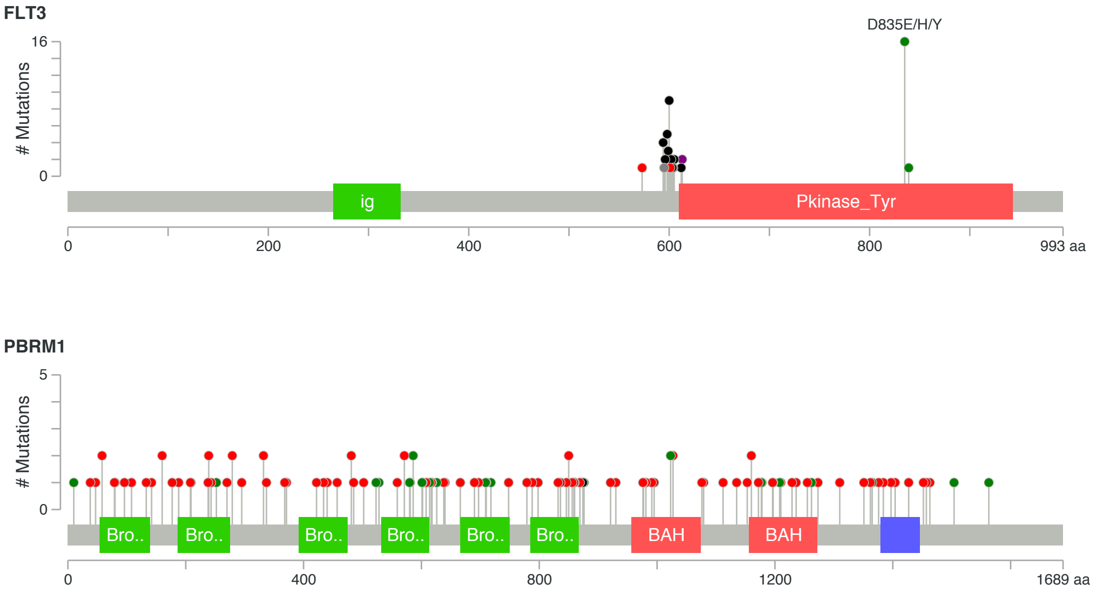

Supplementary Figure 5

Somatic mutations in *FLT3* and *PBRM1*. Two examples of driver genes missed by methods using a single signature of tumor evolution. Somatic mutations in *FLT3* and *PBRM1* in Pancan12 are visualized as loliplots. *FLT3* has a recurrent mutation in position 835 that is scored as medium damage by MutationAssesor and hence is missed by the functional damage bias-based method OncodriveFM. *PBRM1* has loss of function somatic mutations distributed along multiple domains of the gene and is missed by the clustering based method OncodriveClust. Red dots represent nonsynonymous mutations, green represents nonsense mutations, and black represents one base pair indel mutations. Figure was made using mutationmapper[6].

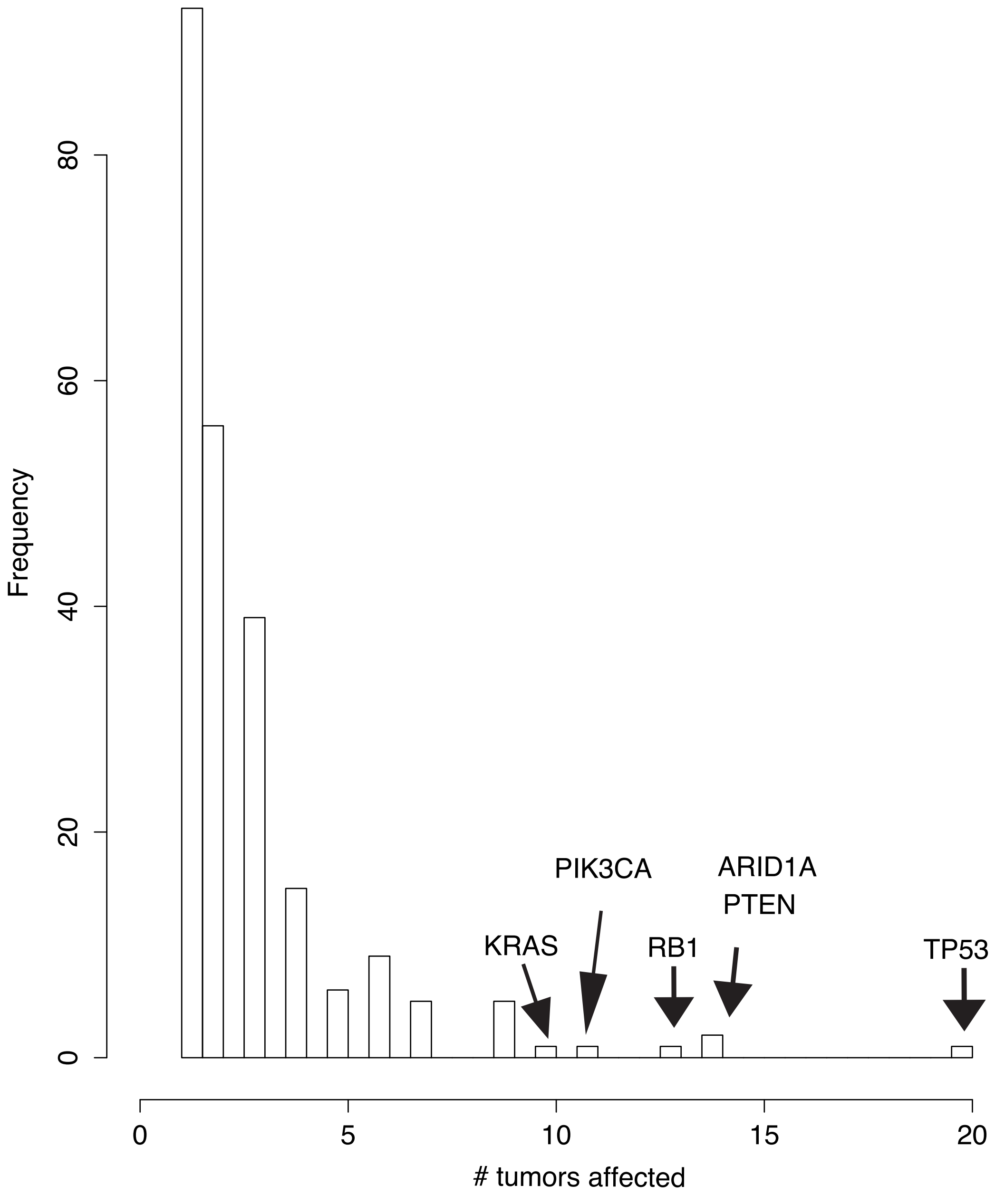

Supplementary Figure 6

Distribution of genes affecting tumor types. Histogram of high confidence driver genes and the number of tumor types affected by them.

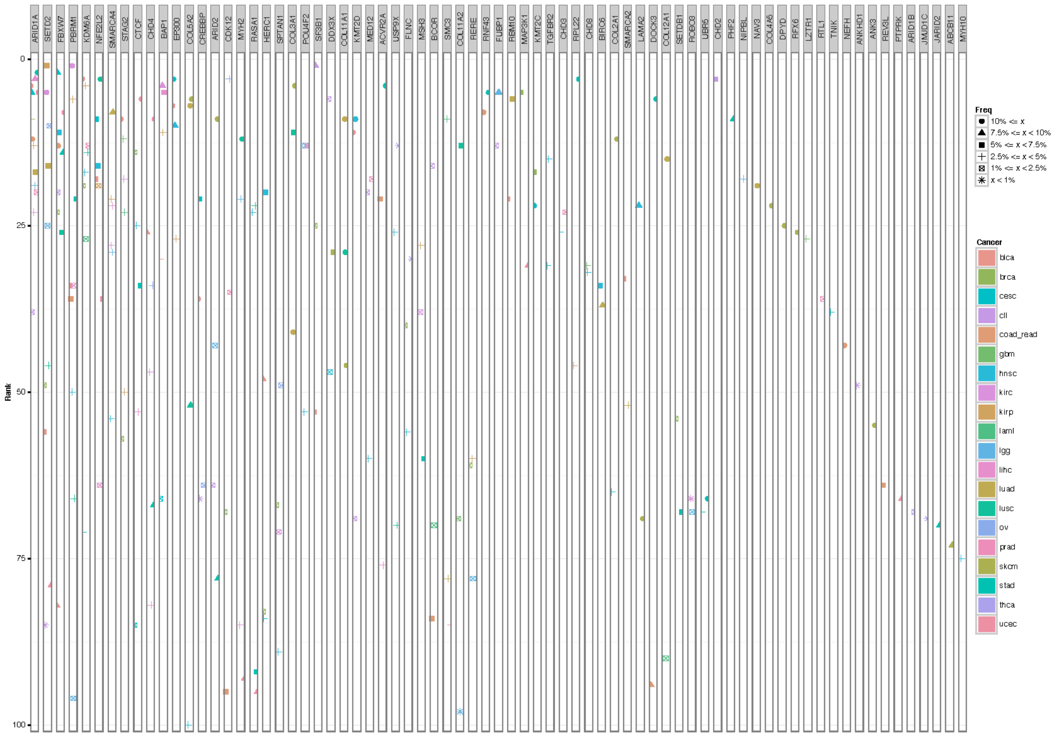

Supplementary Figure 7

Extended figure of the “tumor type – driver gene” connection landscape. Thirty selected genes are shown together with the tumors affected by them, the ranking in that tumor type, and the frequency of patients having the gene mutated.

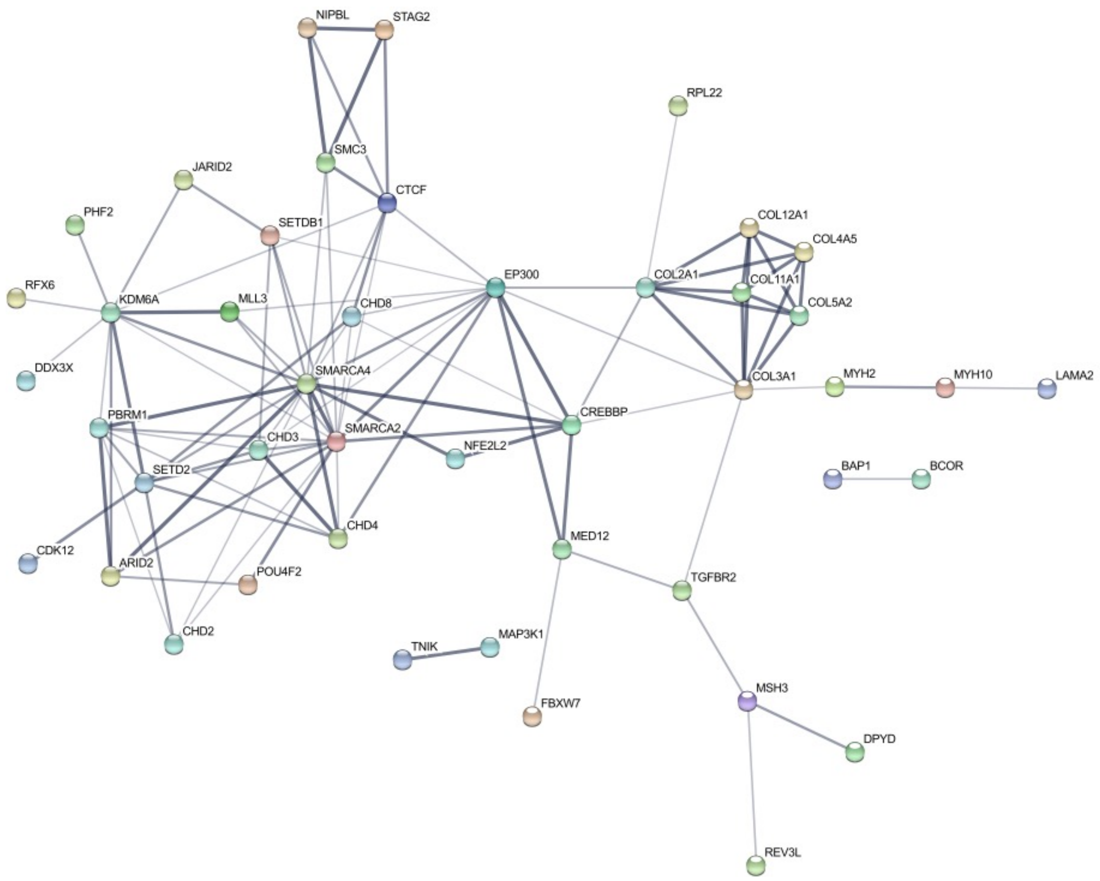

Supplementary Figure 8

STRING PPI analysis of selected genes. STRING enrichment analysis using all functions except text mining shows a significant enrichment for interactions in the unreported TTDG dataset. The main function revealed in these genes is chromatin modification.

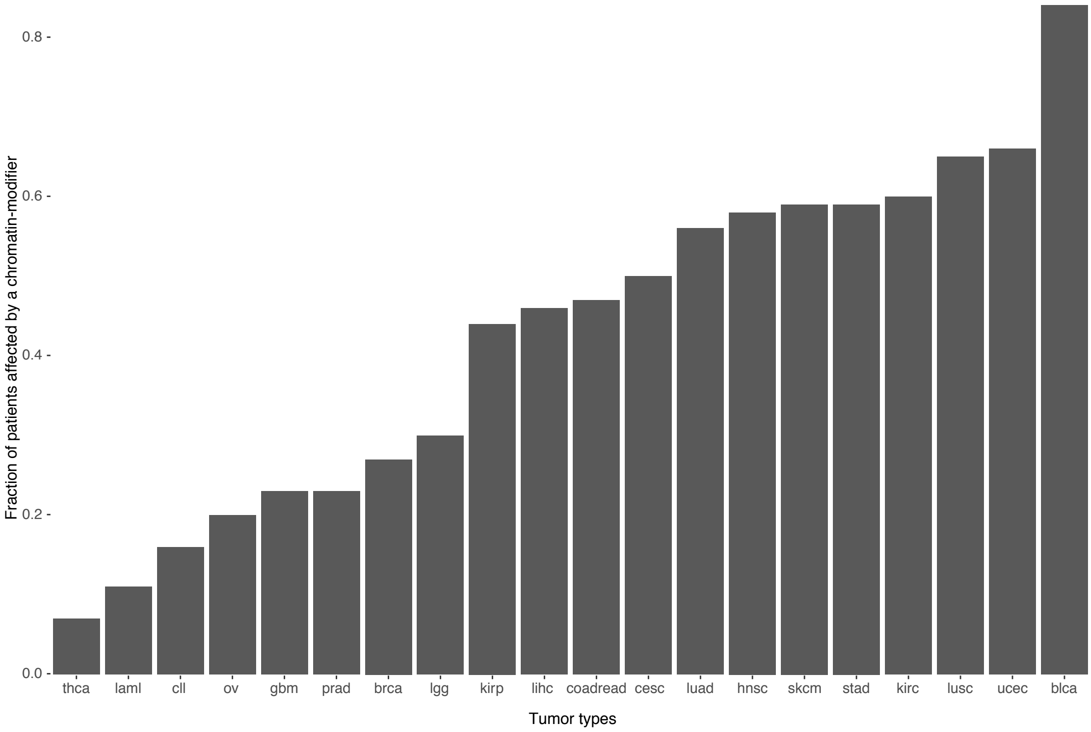

Supplementary Figure 9

Chromatin modifiers affect a large proportion of individuals with cancer. Proportion of individuals harboring a nonsilent mutation in at least one of the novel chromatin modifiers described in the text.

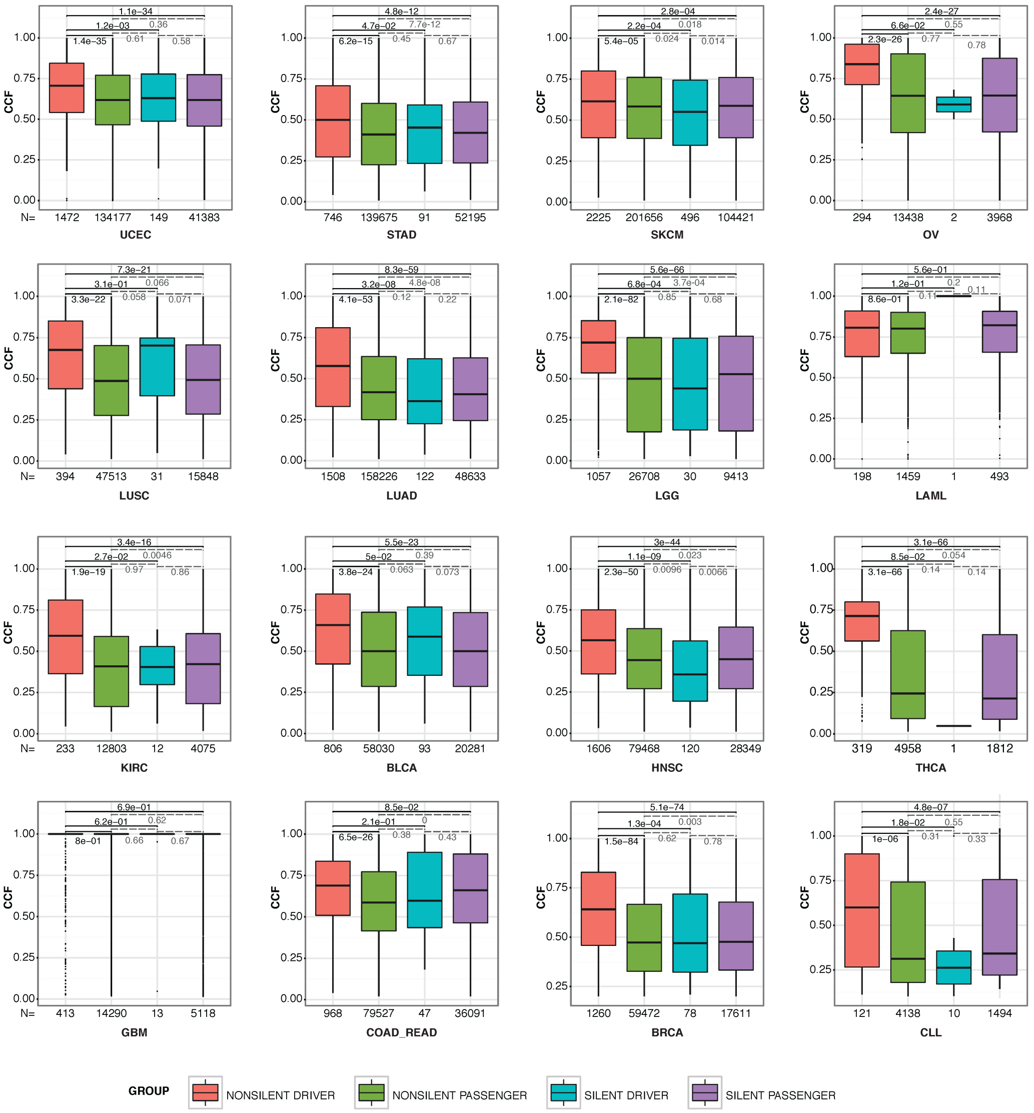

Supplementary Figure 10

CCF distribution of somatic mutations for multiple tumor types. The distribution of CCF values for nonsilent and silent driver mutations and for nonsilent and silent passenger mutations. The P-values shown represent the Wilcoxon-MannWhitney statistical test. The x-axis shows the number of variants used to calculate each distribution, the y-axis shows the CCF.

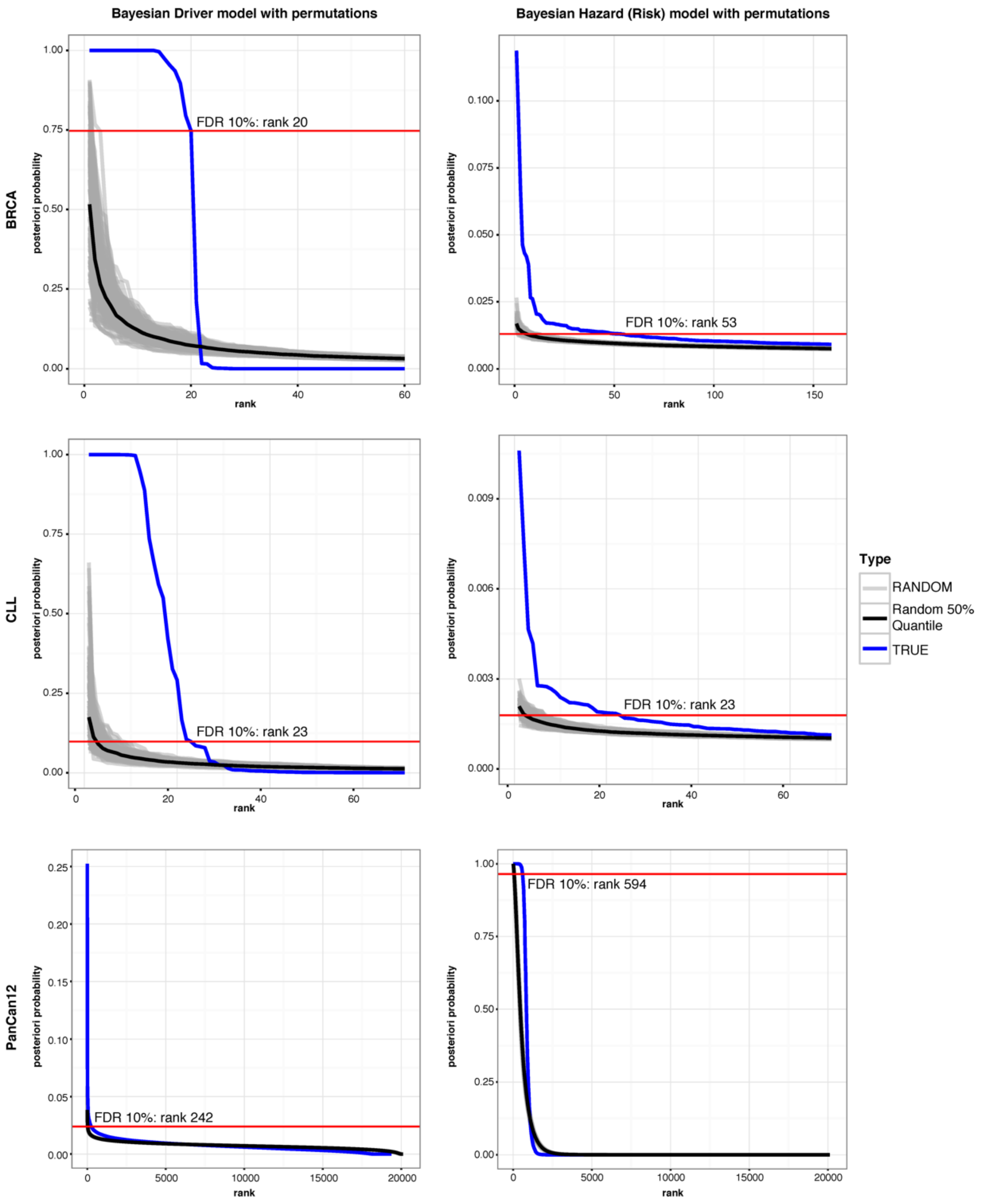

Supplementary Figure 11

Cut off selection for BRCA, CLL, and Pancan12. A randomization procedure reflects the behavior of the posterior probabilities for both cDriver Bayesian models under the null/background model. We obtained a rank cut off where the false discovery rate is less than 10% by comparing test versus null case.

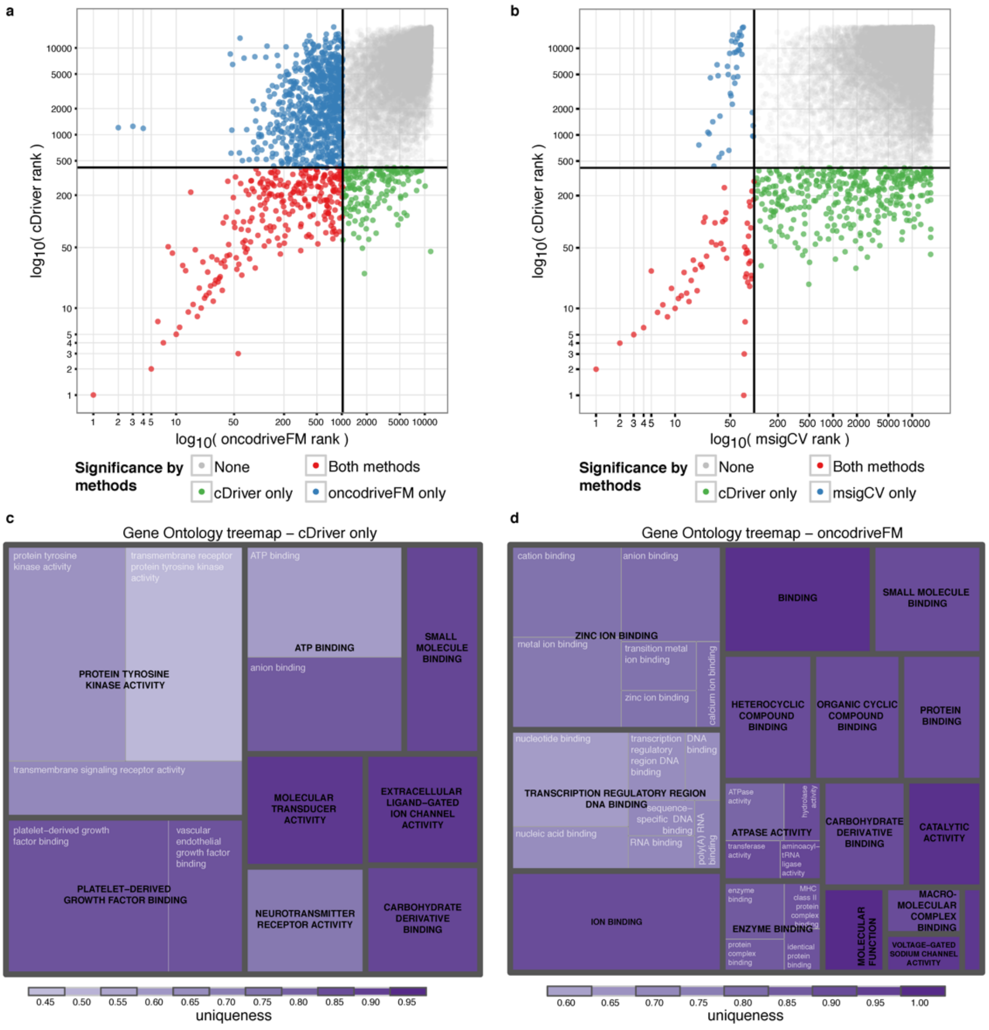

Supplementary Figure 12

Rank-rank plot for cDriver, mutsigCV, and oncodriveFM. a) Rank-rank plot in logarithmic scale for cDriver versus oncodriveFM. b) Rank-rank plot in logarithmic scale for cDriver versus mutsigCV. c) Treemap of functional enrichment of GO terms (molecular function) for significant genes identified only by cDriver (CCF). d) Treemap of functional enrichment of GO terms (molecular function) for significant genes identified only by oncodriveFM. genes identified by mutsigCV only do not show a significant enrichment.

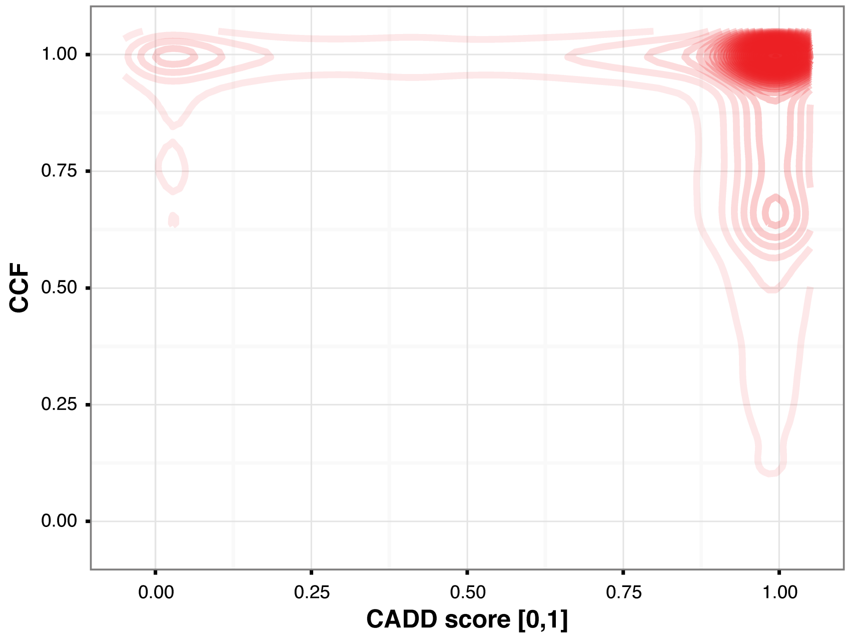

Supplementary Figure 13

Distribution of CCF and functional impact score (CADD) for the genes considered as drivers in at least one of the five gold standards used throughout the manuscript. If a gene in one sample has two mutations, maximum values for both scores are plotted.

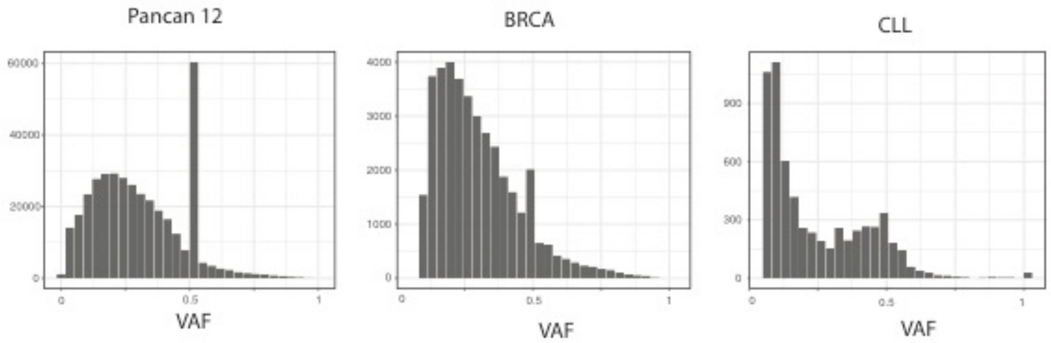

Supplementary Figure 14
Variant allele frequency of somatic calls for Pancan12, BRCA, and CLL.

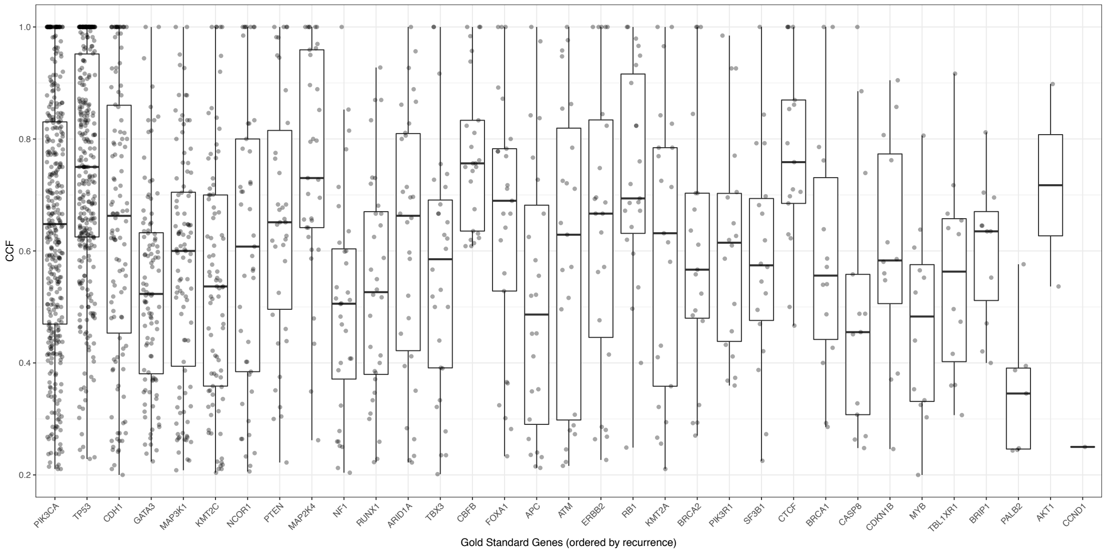

Supplementary Figure 15
CCF distribution of variants found in BRCA gold standard driver genes

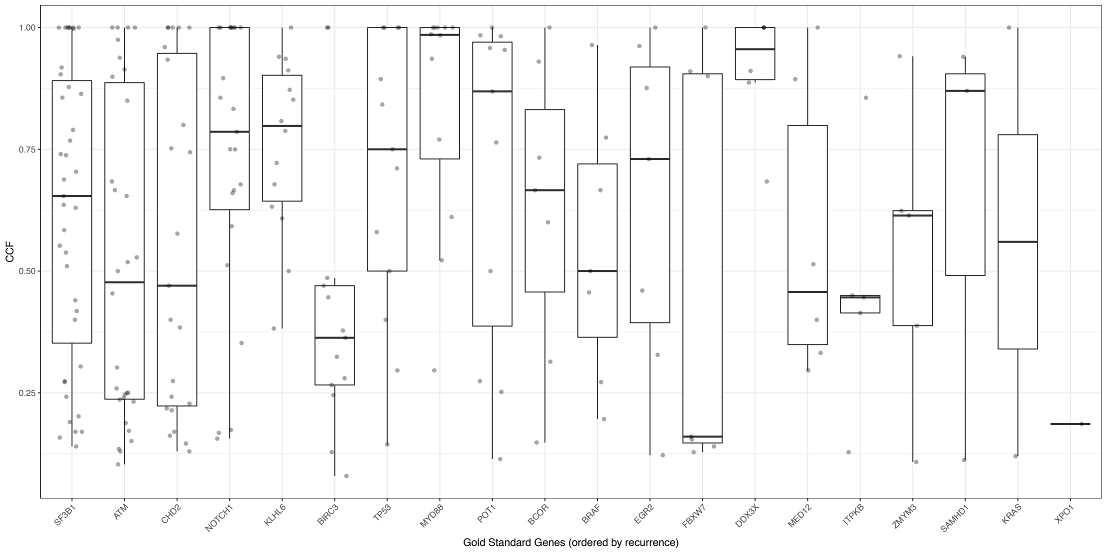

Supplementary Figure 16
CCF distribution of variants found in CLL gold standard driver genes

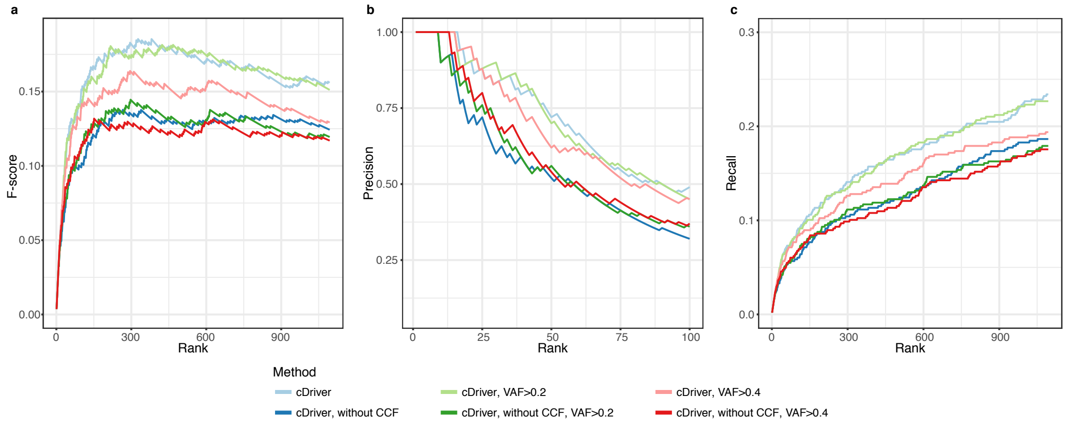

Supplementary Figure 17

Performance in terms of a) Fscore, b) Precision, and c) Recall of cDriver when using the CCF signature compared to not using the CCF signature in three versions of the Pancan12 benchmark set: i/ full, ii/ filter variants with VAF < 0.2, iii/ filter variants with VAF < 0.4. In the full dataset a substantial gain in performance is observed when using the CCF signature (light blue vs. light red). When filtering all subclonal variants (VAF < 0.4) the advantage of using the CCF signature disappears (dark blue vs. dark red).

Supplementary Tables

Supplementary Table 1. High quality datasets for benchmarking

Abbreviation	Tumor type	# Patients	Nonsilent	Silent	Incidence	Source	# Gold standard genes
**BRCA**	Breast invasive carcinoma	762	29929	8612	0,00125	Kandoth et al 2012	33
**CLL**	Chronic Lymphocytic Leukemia	385	8145	3015	0,00005	ICGC	22
**Pancan12***	Pooled set of 12 cancers	3205	291129	90884	0,00450	Kandoth et al 2012	*CGC

Supplementary Table 2. Manually curated gold standard genes for BRCA and CLL

BRCA	CLL
AKT1	ATM
APC	BCOR
ARID1A	BRAF
ATM	CHD2
BRCA1	DDX3X
BRCA2	EGR2
BRIP1	FBXW7
CASP8	ITPKB
CBFB	KLHL6
CCND1	KRAS
CDH1	MED12
CDKN1B	MYD88
CTCF	NOTCH1
ERBB2	NRAS
FOXA1	POT1
GATA3	SAMHD1
KMT2A	SF3B1
KMT2C	TP53
MAP2K4	XPO1
MAP3K1	ZMYM3
MYB	BIRC3
NCOR1	MYC
NF1	
PALB2	
PIK3CA	
PIK3R1	
PTEN	
RB1	
RUNX1	
SF3B1	
TBL1XR1	
TBX3	
TP53	

Supplementary Table 3. F-score, Precision, and Recall at significance level for each gold standard

Method	Significance level position	F-score at significance level	Precision at significance level	Recall at significance level	Max F-score	Max F-score position
**CGC gold standard (547 genes)**						
cDriver*#@	418	0.1803	0.2081	0.159	0.1854	327
msigCV	100	0.1391	0.45	0.0823	0.1806	184
Music	2175	0.0794	0.0497	0.1974	0.0985	793
oncoClust	282	0.1013	0.1489	0.0768	0.1094	660
oncoFM	1025	0.1565	0.12	0.2249	0.1911	353
**Tamborero gold standard (291 genes)**						
cDriver#	418	0.3357	0.2847	0.4089	0.3796	257
msigCV	100	0.2967	0.58	0.1993	0.3424	188
Music	2175	0.1233	0.0699	0.5223	0.1987	806
oncoClust*	282	0.2129	0.2163	0.2096	0.2345	178
oncoFM@	1025	0.272	0.1746	0.6151	0.4073	254
**Kandoth gold standard (127 genes)**						
cDriver	418	0.2936	0.1914	0.6299	0.5134	60
msigCV*#	100	0.4405	0.5	0.3937	0.4585	126
Music	2175	0.0999	0.0529	0.9055	0.25	145
oncoClust	282	0.1858	0.1348	0.2992	0.2533	102
oncoFM@	1025	0.151	0.0849	0.685	0.5226	72
**Lawrence gold standard (260 genes)**						
cDriver@	418	0.3009	0.244	0.3923	0.3878	132
msigCV#	100	0.3167	0.57	0.2192	0.3571	188
Music	2175	0.0986	0.0552	0.4615	0.1778	145
oncoClust*	282	0.1845	0.1773	0.1923	0.2045	180
oncoFM	1025	0.193	0.121	0.4769	0.3567	183
**Xie gold standard (556 genes)**						
cDriver*#@	418	0.2444	0.2847	0.214	0.2578	282
msigCV	100	0.1768	0.58	0.1043	0.2296	228
Music	2175	0.1399	0.0878	0.3435	0.1814	1054
oncoClust	282	0.1313	0.195	0.0989	0.1477	676
oncoFM	1025	0.2011	0.1551	0.286	0.2549	370

* - best abs(Max F-score – F-score at significance level)
# - best F-score at significance level
@ - best maximum F-score

Supplementary Table 6. Number of significant genes per tumor type under FDR10%

Tumor type	Genes
**blca**	29
**brca**	33
**cesc**	74
**cll**	22
**coad_read**	107
**gbm**	42
**hnsc**	27
**kirc**	19
**kirp**	34
**laml**	23
**lgg**	17
**lihc**	54
**luad**	38
**lusc**	24
**ov**	5
**prad**	51
**skcm**	84
**stad**	151
**thca**	3
**ucec**	22

1.Kandoth, C., et al. Mutational landscape and significance across 12 major cancer types. *Nature* **502,** 333-9 (2013).doi:10.1038/nature12634
2.Lawrence, M. S., et al. Discovery and saturation analysis of cancer genes across 21 tumour types. *Nature* **505,** 495-501 (2014).doi:10.1038/nature12912
3.Tamborero, D., Gonzalez-Perez, A. and Lopez-Bigas, N. OncodriveCLUST: exploiting the positional clustering of somatic mutations to identify cancer genes. *Bioinformatics* **29,** 2238-2244 (2013).
4.Futreal, P. A., et al. A census of human cancer genes. *Nat Rev Cancer* **4,** 177-83 (2004).doi:10.1038/nrc1299
5.Xie, M., et al. Age-related mutations associated with clonal hematopoietic expansion and malignancies. *Nat Med* **20,** 1472-8 (2014).doi:10.1038/nm.3733
6. Vohra, S. and Biggin, P. C. Mutationmapper: a tool to aid the mapping of protein mutation data. PLoS One **8**, e71711 (2013).doi:10.1371/journal.pone.0071711
