# Supplementary material for: Signatures of positive selection reveal a universal role of chromatin modifiers as cancer driver genes
Source: Sci Rep. 2017 Oct 13;7:13124. doi: 10.1038/s41598-017-12888-1 (PMC5640613; doi:10.1038/s41598-017-12888-1)
Supplement: Supplementary file 2 — Supplementary Notes [file 41598_2017_12888_MOESM2_ESM.pdf]

Supplementary notes

Signatures of positive selection reveal a universal role of chromatin modifiers as cancer driver genes

Luis Zapata, Hana Susak, Oliver Drechsel, Marc R. Friedländer, Xavier Estivill and Stephan Ossowski

CCF calculation

We have developed a function for estimation of Cancer Cell Fraction (CCF) as part of the cDriver package. We provide a simplified CCF calculation function, in which we do not reconstruct the clonal structure, but estimate CCF independently for each mutation. To this end we focused on the estimation of the fraction of cells carrying a mutation of interest independently of the number of clones. cDriver's Bayesian model for cancer driver gene prediction can work with any model for CCF prediction. However, algorithms for predicting the clonal structure are usually designed for deep-sequencing or whole-genome data and their running time is long compared to cDriver's CCF model (e.g. 3 days for a test case of 385 CLL patients using PyClone (PMID: 24633410). Here we describe the development of cDriver's CCF formula in detail.

To simplify calculation of CCF without inferring the actual clonal structure we made several assumptions: a) a somatic point mutation should be observed in approximately half of the reads in case of a heterozygous variant in a diploid locus affecting all tumor cells, b) all somatic variants are heterozygous (i.e. only one allele is affected by the same mutation per cell), c) for each mutation we consider only three populations of cells (affected and non-affected tumor cells and normal cells), i.e. we use a 2-population model with normal admixture, d) a mutation at a specific position of the genome only happens once during the evolution of the tumor and cannot be reversed.

Fig. 1 shows an example of two cancer subpopulations 1) dark blue with red circle mutation and 2) light blue that evolved from dark blue population by acquiring blue star mutation (keeping red circle mutation). After aligning reads from the sequenced tumor sample to the reference genome we can directly calculate variant allele frequency (VAF) for each observed mutation as the fractions of reads showing the alternative allele. In the diploid regions, we can assume that VAF value is half of the cancer cell fraction CCF if we have a perfect separation of tumor and normal cells, i.e. purity of 1.

The example shown in Figure 1b) extends the model to allow for purity of tumor samples bellow one (i.e. some normal cells are sequenced together with tumor cells). Therefore, the

total amount of reads without somatic mutations will be higher, and the VAF value will underestimate CCF. To obtain purity-corrected CCF we need to divide by purity. Tumor sample purity can either be estimated from histopathology or intrinsically using tools like ABSOLUTE (ref. doi:10.1038/nbt.2203). Figure 1b shows the model with two cancer subpopulations and one normal population. As cDriver-CCF considers each mutation separately this model always applies. Therefore, in a diploid region we can calculate CCF for each mutation as:

$$CCF_{SNV} = \frac{VAF_{snv} * 2}{Purity} \quad (1)$$

However, this formula will not hold if a copy number change overlapping the somatic point mutation is identified, i.e. if all or a fraction of tumor cells is not diploid at the focal point mutation. To interrogate how the correlation of VAF and CCF for point mutations changes with growing fractions of cells affected by a CNV at the same locus we simulated deletions (Fig. 2) and copy gains (Fig.3) at variable CCF. We introduce two new parameters, $\text{ploidy}_{\text{CNV}}$, describing the allele number in cells affected by the CNV (e.g. 1 for deletion, 3 for duplication) and CCF_{CNV}, describing the estimated fraction of cancer cells carrying the copy-number change. In order to efficiently calculate the CCF of point mutations taking into account CNVs of variable CCF we made the following assumptions, in addition to the assumptions discussed above: a) only one heterozygous CNV event per locus, b) CNVs occur only once during the evolution of a tumor and cannot be reversed, c) normal cells have a ploidy of 2. Note that due to these assumptions the results are not affected if the CNV is present in the same subpopulation as the point mutation or in other.

As shown in figures 2 and 3 VAF_{SNV} and CCF_{SNV} are linearly related (panels a and b) with intercept equal to zero, but with varying slope. Further we noticed that CCF_{CNV} is linearly related to the ratio of CCF_{SNV} and VAF_{SNV} (panels c) when we fix the ploidy of the CNV (e.g. deletion or duplication). Intercept (β_0) for this linear relation (panels c) is always equal to 2, and slope (β_1) is equal to $\text{ploidy}_{\text{CNV}}$ - 2. This also holds for higher ploidies than 3 (figures not included). To conclude, if the values for ploidy of CNV-affected cells ($\text{ploidy}_{\text{CNV}}$), the fraction of cells affected by CNVs (CCF_{CNV}), the variant allele frequency of the SNV in the read alignment (VAF_{SNV}), and the tumor purity are known parameters, we can calculate CCF in any ploidy status, except in loci affected by multiple overlapping CNVs in different subpopulations. The generalized CCF_{SNV} equation for SNVs and indels in diploid and non-diploid loci is then:

$$CCF_{SNV} = \frac{VAF_{SNV} * (2 + (ploidy_{CNV} - 2) * CCF_{CNV})}{Purity} \quad (2)$$

where VAF_{SNV} is the observed variant allele frequency, ploidy_{CNV} is the ploidy of cells affected by an overlapping CNV, CCF_{CNV} is the fraction of cells affected by the CNV, and purity represents the fraction of tumor cells in the sequenced sample.

The values for CCF_{CNV} and ploidy_{CNV} can be obtained using any tool for somatic CNV prediction that also reports the cancer cell fraction of CNVs. Purity values can be obtained from clinical data, cell sorting efficiency, or calculated intrinsically by e.g. ABSOLUTE. Purity data for most TCGA samples can be obtained from the TCGA clinical data repository.

For cases with a complex ploidy pattern (e.g. whole genome duplications, chromothripsis, large fraction of tumor genome affected by CNVs) cDriver allows the use of CCF estimates from tools using a more complex model of clonal structure and tumor evolution, e.g. Pyclone. However, we found that the ranked driver gene lists predicted using CCF of cDriver vs. cellular prevalence of PyClone showed a spearman correlation of 0.93 and a Kendall tau distance of 0.097 (Figure 4), i.e. the same driver genes are predicted with marginal changes in absolute rank. To obtain cellular prevalence using PyClone for 383 CLL patients required more than 2 days (parameters set as suggested by authors), while cDriver's CCF function finished in less then a second (0.041 s) on the same architecture.

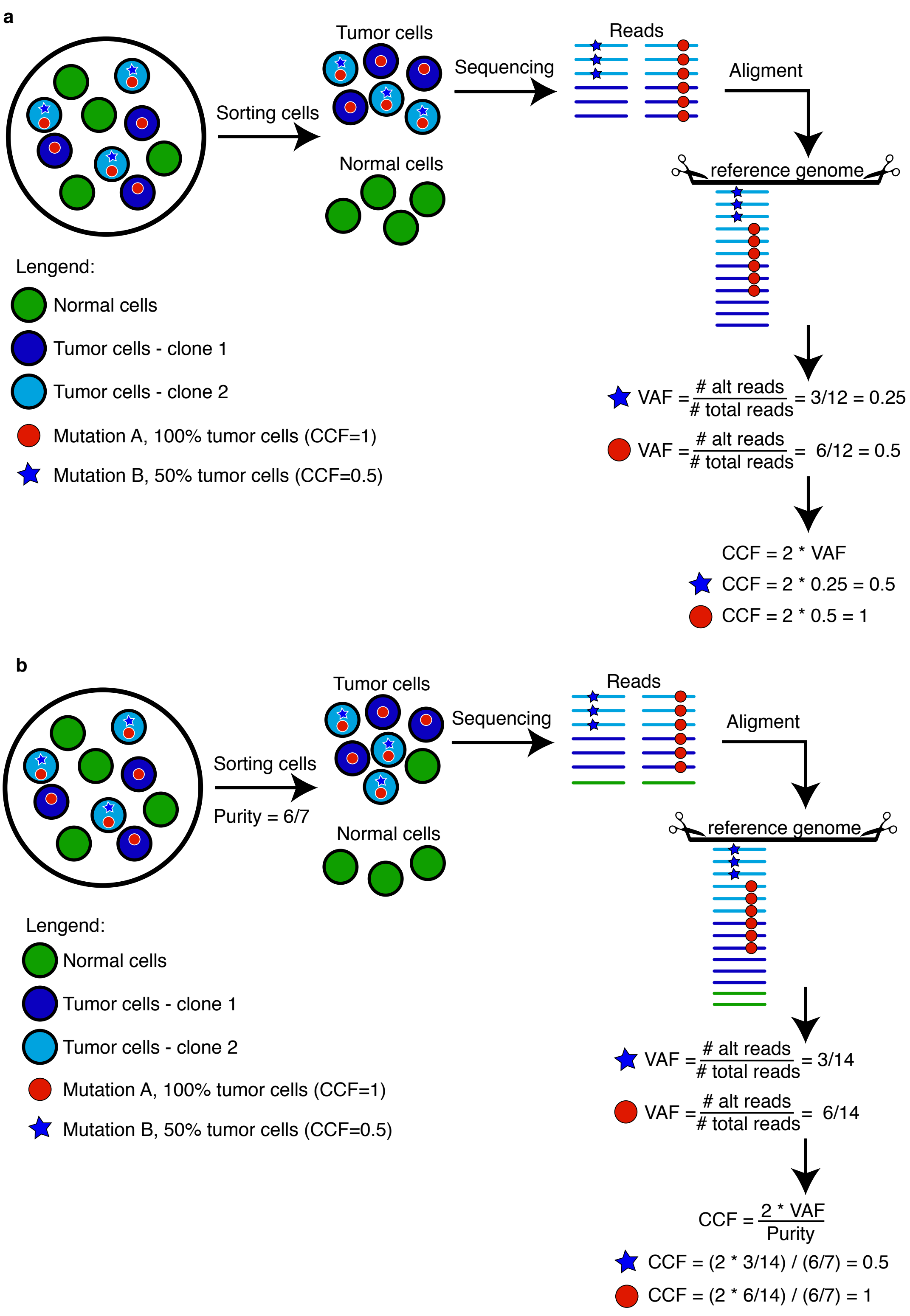

Figure 1. Schematic model for CCF-inference from read counts in diploid regions when purity is 100% (a) and when purity is less than 100% (b).

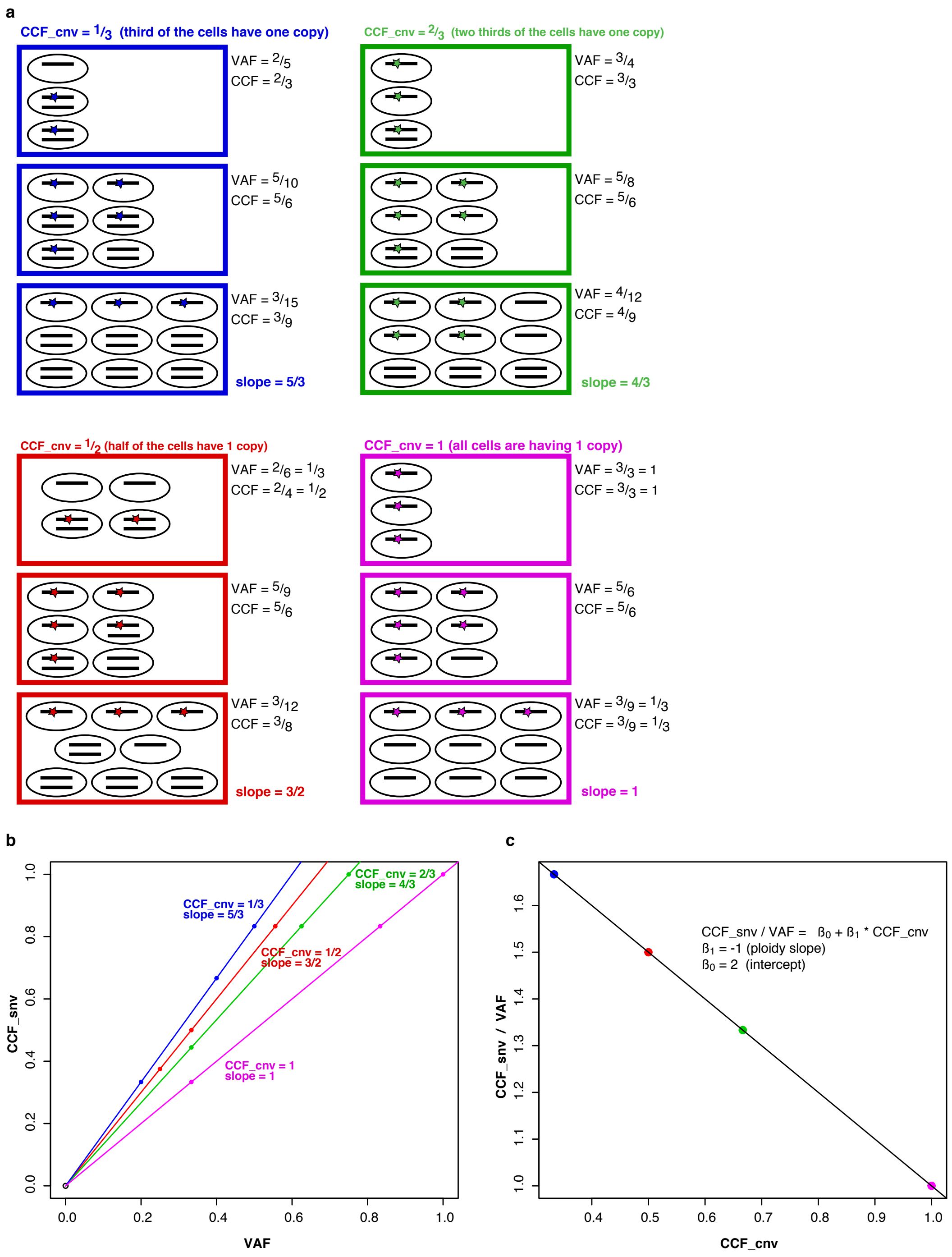

Figure 2. Toy model describing the calculation of CCF_{SNV} from VAF_{SNV} if a fraction of cancer cells has a hemizygous deletion (ploidy 1). In figures a and b the fraction of cells affected by a deletion (CCF_{CNV}) is varied. Figure b shows the dependence of CCF_{SNV} from VAF_{SNV} at different CCF_{CNV}. Figures c shows the relationship between CCF_{SNV}/VAF_{SNV} and CCF_{CNV}.

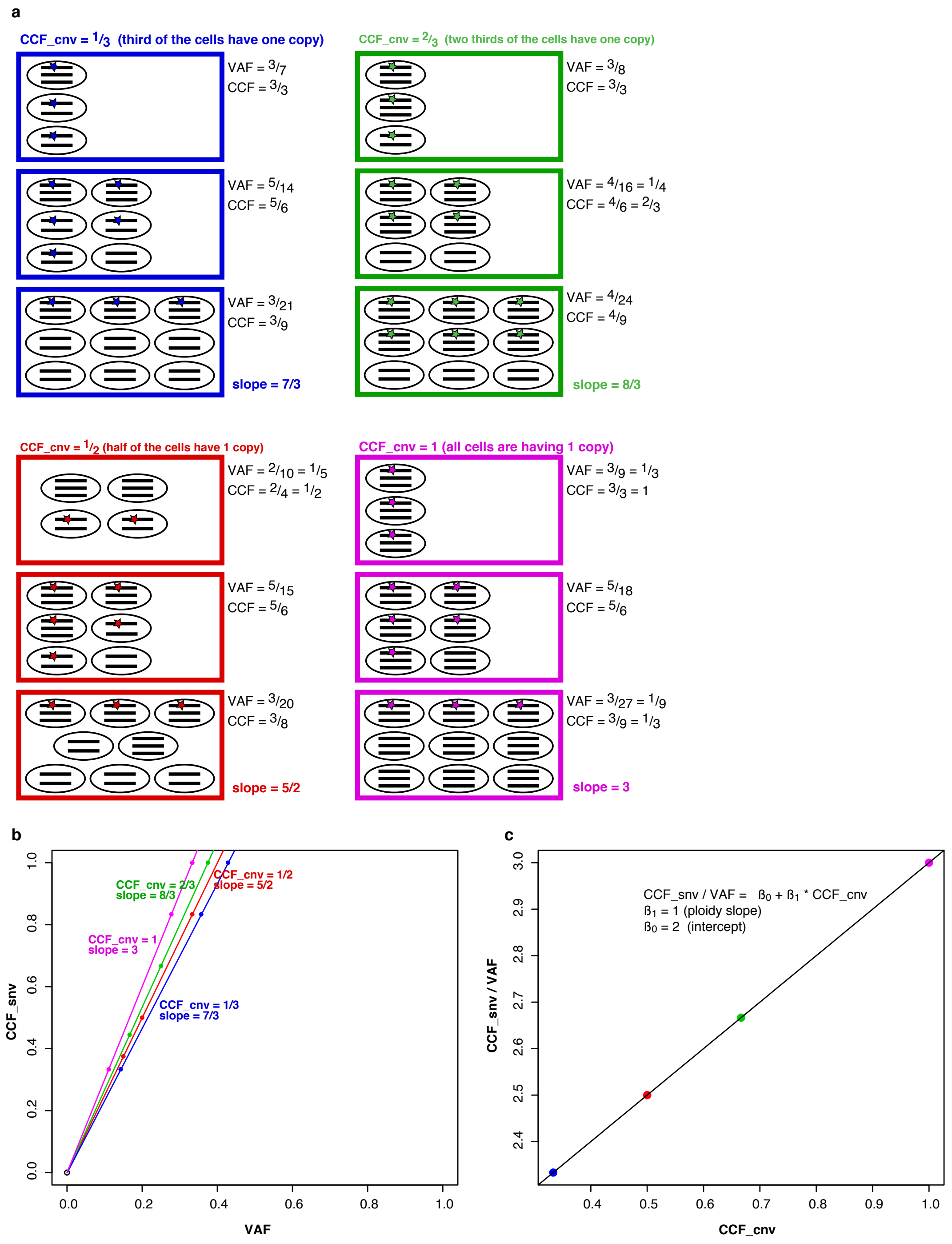

Figure 3. Toy model describing the calculation of CCF_{SNV} from VAF_{SNV} if a fraction of cancer cells has a hemizygous copy gain (ploidy 3). In figures a and b the fraction of cells affected by a copy gain (CCF_{CNV}) is varied. Figure b shows the dependence of CCF_{SNV} from VAF_{SNV} at different CCF_{CNV}. Figures c shows the relationship between CCF_{SNV}/VAF_{SNV} and CCF_{CNV}.
